# Comparing the content and quality of video, telephone, and face-to-face consultations: a non-randomised, quasi-experimental, exploratory study in UK primary care

**DOI:** 10.3399/bjgp19X704573

**Published:** 2019-07-02

**Authors:** Victoria Hammersley, Eddie Donaghy, Richard Parker, Hannah McNeilly, Helen Atherton, Annemieke Bikker, John Campbell, Brian McKinstry

**Affiliations:** Usher Institute of Population Health Sciences and Informatics;; Usher Institute of Population Health Sciences and Informatics;; Usher Institute of Population Health Sciences and Informatics;; Medical Teaching Unit, University of Edinburgh, Edinburgh.; Warwick Medical School, University of Warwick, Coventry.; Usher Institute of Population Health Sciences and Informatics;; General Practice and Primary Care, University of Exeter Medical School, University of Exeter, Exeter.; Usher Institute of Population Health Sciences and Informatics;

**Keywords:** communication, general practice, health services research, telemedicine

## Abstract

**Background:**

Growing demands on primary care services have led to policymakers promoting video consultations (VCs) to replace routine face-to-face consultations (FTFCs) in general practice.

**Aim:**

To explore the content, quality, and patient experience of VC, telephone (TC), and FTFCs in general practice.

**Design and setting:**

Comparison of audio-recordings of follow-up consultations in UK primary care.

**Method:**

Primary care clinicians were provided with video-consulting equipment. Participating patients required a smartphone, tablet, or computer with camera. Clinicians invited patients requiring a follow-up consultation to choose a VC, TC, or FTFC. Consultations were audio-recorded and analysed for content and quality. Participant experience was explored in post-consultation questionnaires. Case notes were reviewed for NHS resource use.

**Results:**

Of the recordings, 149/163 were suitable for analysis. VC recruits were younger, and more experienced in communicating online. FTFCs were longer than VCs (mean difference +3.7 minutes, 95% confidence interval [CI] = 2.1 to 5.2) or TCs (+4.1 minutes, 95% CI = 2.6 to 5.5). On average, patients raised fewer problems in VCs (mean 1.5, standard deviation [SD] 0.8) compared with FTFCs (mean 2.1, SD 1.1) and demonstrated fewer instances of information giving by clinicians and patients. FTFCs scored higher than VCs and TCs on consultation-quality items.

**Conclusion:**

VC may be suitable for simple problems not requiring physical examination. VC, in terms of consultation length, content, and quality, appeared similar to TC. Both approaches appeared less ‘information rich’ than FTFC. Technical problems were common and, though patients really liked VC, infrastructure issues would need to be addressed before the technology and approach can be mainstreamed in primary care.

## INTRODUCTION

Communication technologies are central to contemporary life, yet, with the exception of the telephone, they are not routinely used for communication between clinician and patient.^1^ The use of telephone consultation (TC) as an alternative to face-to-face consultation (FTFC) has become widespread in many general practices, partly because it is perceived to be more time efficient and can alleviate primary care access problems for housebound patients or those who work.^2^ However, research has shown that, compared with FTFCs, TCs are shorter, cover fewer problems, include less data gathering, less advice and rapport building, and are perceived to be suitable only for uncomplicated presentations, are less safe, and may not save time.^3^^,^^4^ Lack of informal visual examination of the patient to gauge how generally unwell the patient is, and the gathering of non-verbal clues, were considered important features of FTFC lost in TCs.^2^

Internet-based video consulting has the potential to overcome this barrier, particularly for conditions that do not routinely require contact examination, for example, mental health review and assessment of inhaler technique, while potentially improving access and time efficiency, especially for patients who work during surgery hours. The authors’ previous research^2^ and that of others^5^ also shows that attendance at a surgery with the associated sacrifice of time and convenience for the patient comes with an expectation of a ‘reasonable’ time to be spent in the consultation almost regardless of the complexity of the presenting problem, whereas it is recognised that brief TCs (where little attendant inconvenience has been incurred) are acceptable for similar presenting problems. It is possible that similar expectations will surround uncomplicated video consultations (VCs), allowing them to be briefer, and potentially saving clinician time.

The increasing popularity of video-over-internet programmes such as Skype, and growing demands on primary care services, have led to calls from governments and health service planners for secure versions of these technologies to be adopted in general practice.^6^^,^^7^ The most recent *NHS Long Term Plan* mandates the availability of online services such as VC within the next 5 years.^7^ In addition, there is evidence of patient demand for such a service,^8^^,^^9^ and increasing provision from the private sector. However, very few NHS practices have adopted it.^10^ Moreover, many unanswered questions remain about the content, quality, and appropriateness of VC for different conditions and patients.^11^^,^^12^

In tandem with a Scottish Government pilot of VC, using the web-based platform Attend Anywhere^13^ in various clinical environments, this study explored the use of VC in general practice to determine its acceptability to clinicians and patients, and to examine how VCs varied from FTFCs and TCs in terms of length, quality, and content. The researchers focused on follow-up consultations because physical examination, and therefore an FTFC, was less likely to be required in a follow-up consultation than in an acute first consultation, and to provide time for consent and familiarisation with the system. A content analysis of VCs, TCs, and FTFCs and the results of a post-consultation satisfaction questionnaire are presented in this study. The authors report qualitative findings, examining the views of participant patients and clinicians in a companion article.^14^

**Table:** How this fits in

In many countries policy drives are implemented to introduce video consultation (VC) to improve access to care; however, how it compares, in terms of length, content, and quality, with face-to-face (FTFC) and telephone (TC) consultations in a primary care setting remains unknown. This research is the first to use audio-recordings of follow-up consultations in all three modes to explore these issues. VC was popular with those who used it. VC length was similar to TC and both were considerably shorter than FTFC; however, VC and TC dealt with fewer problems, demonstrated fewer instances of information giving by clinicians, and scored less well on a range of consultation-quality items. Though there are potential advantages for people who work, or who have mobility or mental health problems, the introduction of VC in primary care needs to be conducted carefully in a strong evaluative framework.

## METHOD

### Consent

Patient consent for the follow-up consultation to be audio-recorded and analysed was obtained via Online Surveys (https://www.onlinesurveys.ac.uk/) or as written consent before some FTFCs were recorded. Clinicians provided written consent at the start of the study.

### Setting

The study was conducted in general practices in Scotland. The NHS in Scotland provides care for free, based on need, and funded by general taxation. GPs are remunerated on a capitation basis regardless of consultation rate or mode.

### Participants and sample size

The authors approached practices in Lothian, Scotland, through a local GP newsletter and aimed to recruit 10 clinicians (GPs or practice nurses) from up to five practices.

Patients aged >16 years requiring a follow-up consultation and able to consent were eligible to take part in the study. Participating patients had to have access to an internet-connected computer with a camera and sound capability, tablet, or 4G and/or Wi-Fi-enabled smartphone (running Google Chrome or iOS app), and a working email address. The study size was based on a study by Teare *et al* who recommend a sample size of 70 participants for exploratory studies with continuous outcomes and 60–100 participants for binary outcomes (taking into account that there may be some dropouts and failed consultations).^15^

### Equipment

Very low broadband speeds (in some cases <4 Mb download and 0.4 Mb upload) in NHS systems (average download speed in Scotland is 70 Mb), and a local ban on Google Chrome for security reasons, meant that it was not possible to run Attend Anywhere adequately through the practice NHS computer systems. The authors provided separate high-speed broadband and Wi-Fi to the practices, along with Samsung Galaxy tablets with stands and external speakers, for each participating clinician. Audio-recording was conducted using digital recorders placed in front of the speakers for VCs, beside the phone in speakerphone mode for TC, and on the desk between patient and clinician for FTFC.

### Intervention

**Figure 1.**
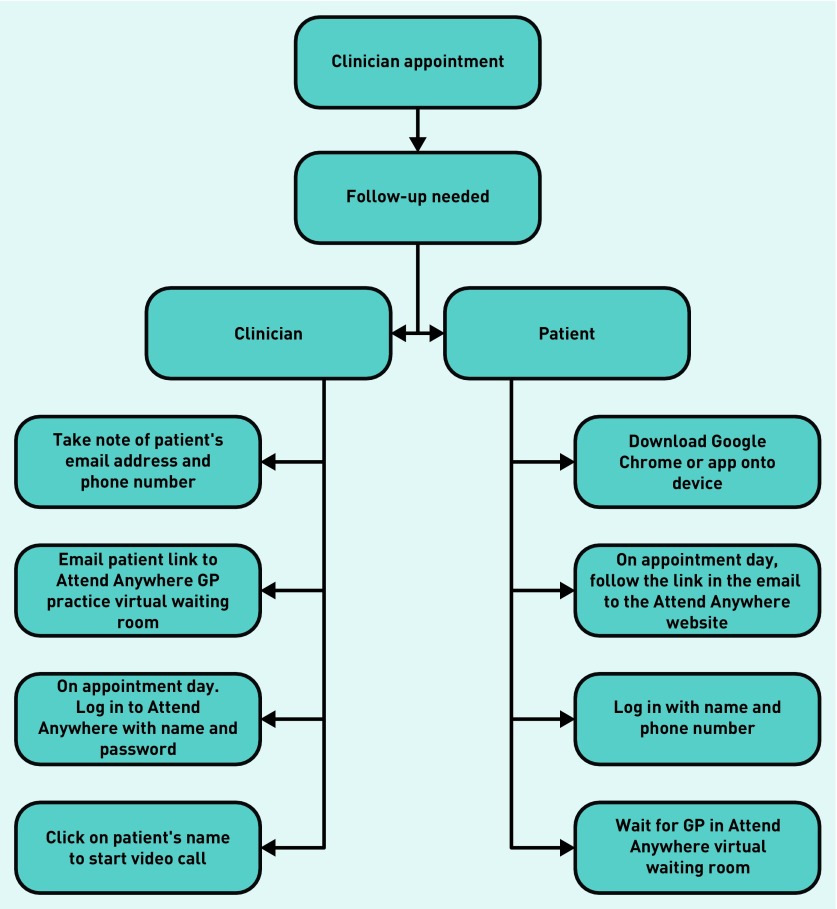
***The process for setting up video consultation for clinicians and patients.***

**Figure 2.**
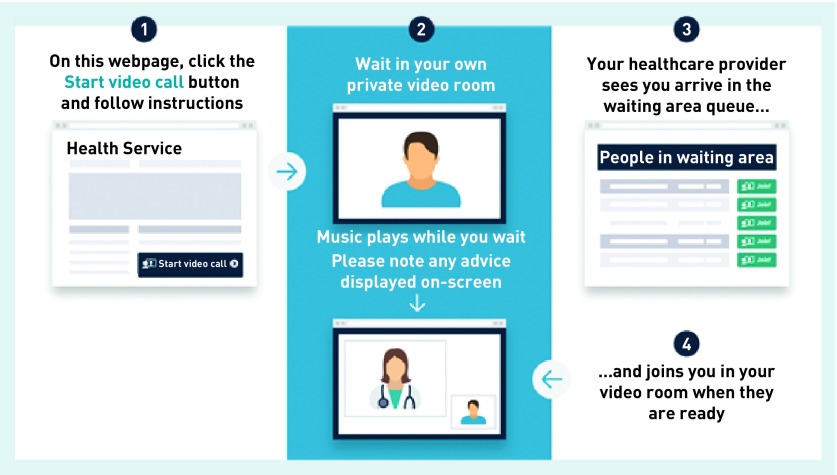
***How patients access Attend Anywhere. Image reproduced with permission.***

The main study intervention was the introduction of VC as an alternative form of follow-up consultation. Figure 1 shows the process of setting up a VC for the clinician and patient, and Figure 2 shows how the patient initiated a consultation. Each clinician aimed to audio-record 5–10 minutes each of VCs, FTFCs, and TCs. Attend Anywhere is an end-to-end fully encrypted VC service. Unlike with Skype and FaceTime, patients are unable to directly call the clinician and instead are placed in a virtual waiting room. To use Attend Anywhere participants required internet access, the Google Chrome web browser on a computer (with a web camera) or Android mobile device, or an app on Apple iPads or iPhones. Patients recruited to have a VC were emailed a secure web link with the date and time of their consultation.

### Recruitment of patient participants

Clinicians asked eligible patients whom they planned to see for review if they wished to take part in the study and had the necessary equipment to conduct a VC. If so, they offered the patient a choice of VC, FTFC, or TC for follow-up, and requested permission to record this consultation. A note was taken of the patient’s telephone number and email address, and an appointment made for the follow-up consultation. If there was no response to email, the research team subsequently contacted the patient by email or phone to obtain consent and to explain how the system would work, along with instructions on how to download Google Chrome if they did not already have this. They were then sent an email link to the Attend Anywhere site. Patients were also given the opportunity to test their equipment with the research team. On the day and at the time of the appointment patients logged on and awaited the clinician contact. TCs and FTFCs were conducted in the usual way. Clinicians checked with the patient if they were still happy to have the consultation recorded at the time of the consultation.

### Questionnaires

After the consultation, patients were emailed a link to a consultation-mode-specific, online questionnaire (via Online Surveys) based on the consultation experience questions of the GP Patient Survey.^16^ For TC and VC, there were additional questions about the technical aspects of the encounter and the appropriateness of this consultation mode for similar future problems (questions are available from the authors on request).

### Data collection

Researchers reviewed each recorded consultation and measured the parameters listed in Box 1. The Roter Interaction Analysis System (RIAS)^17^ was used to determine the specific content of the consultations. The RIAS is a well-validated method of consultation analysis of voice recordings, which involves noting the presence of 40 mutually exclusive categories for every utterance in the consultation and is designed to directly reflect the content and context of routine medical dialogue. Two members of the research team had previously used RIAS to compare TC and FTFCs^4^ and provided training for a third. Additionally, they used a previously adapted and validated scoring system^18^ designed by the Royal College of General Practitioners (RCGP) to explore the quality of GP trainee consultations, and this provided the research team with a ‘gold standard’ (Box 1). Analysis was carried out over the course of 2 days, by all of the researchers as a group, to ensure consistency of coding. Demographic details, previous and subsequent GP, and hospital and out-of-hours attendances were collected from patients’ case-notes.

**Box 1.** Types of data collected from recorded consultations and case-note review

**Problem type and lifestyle**	**RCGP quality indicators^18^**	**Roter Interaction Analysis System^17^**	**Workload**
Number of problems raised Number of problems dealt with Types of problem raised Lifestyle advice given	If patients’ own health understanding of their problems is sought If patients’ concerns are sought If there are attempts to place problems in a psychosocial context Explanation of diagnosis Explanation of treatment or treatment options Checking understanding Shared decision making Safety netting (telling patient what to do if condition does not improve or deteriorates)	Social speech (personal remarks, social conversation) Presence of humour (laughs, jokes) The type and amount of key questioning by doctor The type and amount of questioning by patient Balance of open and closed questions Presence of expressed emotion (anger concern) Presence of disapproval, criticism Requests for, and giving of, reassurance Presence of expressed empathy	Consultation length Number of times seen by clinician in last year In the following 28 days, number of: — FTFC, TC, VC appointments — out-of-hours appointments — A&E and hospital admissions

A&E = accident and emergency department. FTFC = face-to-face consultation. RCGP = Royal College of General Practitioners. TC = telephone consultation. VC = video consultation.

### Analysis

Descriptive statistics were calculated for patient demographics (derived from practice records) and the parameters in Box 1. Demographic measures included age, sex, and ethnicity (as recorded in medical record), and socioeconomic status was based on the Scottish Index of Multiple Deprivation (SIMD),^19^ which centres on postcodes, with SIMD 1 referring to the most deprived areas and SIMD 5 to the least deprived areas. In general, for categorical variables, reported frequencies and percentages were reported. Means and standard deviations (SDs) were reported for patient age, consultation length, and number of problems raised. Medians and interquartile ranges (IQR) were reported for number of consultations in the year before the index appointment since the data were skewed. Similarly, for the continuous RIAS scores, the authors reported medians with IQR in addition to arithmetic means if items displayed a skewed distribution. To aid interpretation, exploratory two-sample *t*-tests were performed to compare the telephone/VC groups with FTFC groups, which were supported by median tests. RCGP quality indicators were compared across consultation groups with Fisher’s exact tests calculated using R software (version 3.5.0). SAS software (version 9.4) was used to perform the RIAS analysis. The researchers calculated mean differences and corresponding 95% confidence intervals (CIs) for consultation length. All other analyses were performed using SPSS (version 21.0).

## RESULTS

### Recruitment of clinicians

A total of 12 practices initially expressed interest in participating, and three subsequently declined following a telephone discussion. The researchers visited the remaining nine practices to explain the project, and a further three declined, largely due to fear of increased workload and disruption to practice working. Out of these six, five practices were chosen to represent a mix of small-town, suburban, and city-centre practices. Later in the study a further practice (with three participating GPs) was recruited to boost patient recruitment. Overall, eight male and five female clinicians, aged 29–55 years, took part (details of individual clinician recruitment to the study are available from the authors on request). Two of the clinician participants had regularly used software like Skype/FaceTime in a clinical setting (one in rural Australia and one in a military setting).

### Recruitment of patients

Recruitment took place between June 2017 and September 2018. Most clinicians found it difficult to recruit patients to participate in the study. Table 1 shows the number of patients who initially verbally agreed to participate, those who gave online or written consent to the researchers, and the number of recordings obtained for analysis. Sometimes clinicians forgot to switch on the audio-recorder and this was the main reason for the difference between numbers consenting and the number of recordings made. To improve recruitment, researchers placed themselves in the waiting rooms to explain the study in advance to waiting patients and to provide a reminder to clinicians. Banners advertising the study were placed in surgery waiting areas.

**Table 1.** Patient recruitment and number of recordings

**Patients and recording**	**FTFC**	**TC**	**VC**	**Total**
Patients initially agreeing to participate, *n*	64	71	68	203
Online or written consent obtained, *n*	54	56	52	162
Number of recordings completed^a^	51	53	45	149

a Two patients recorded two consultations. FTFC = face-to-face consultation. TC = telephone consultation. VC = video consultation.

### Characteristics of consenting patients

Participants choosing VC were younger on average, mean 42.0 years (standard deviation [SD] 15.9), compared with those choosing TC (54.3 years, SD 16.8) or FTFC (52.3 years, SD 16.8) and more likely to be female (FTFC 21/54 [39%], TC 31/56 [55%], VC 28/52 [54%]). However, there were more females overall in the younger age group. Video consultors were similar in terms of deprivation as measured by the SIMD^19^ (study sample’s SIMD quintiles are available from the authors on request). Of the 98 participants giving data on ethnicity, the number (%) of white British was 21/27 (78%) for FTFC, 32/34 (94%) for TC, and 32/37 (86%) for VC. Of those providing ethnicity, the vast majority were white British (85/98 [87%]).

Patients in the FTFC group had more consultations in the preceding year (median 10 per year, IQR 5–15) compared to the other groups (median TC = 6 [IQR 4–10], median VC = 5 [IQR 3–9).

### Technical challenges

Although the authors hoped to overcome connection problems by providing additional broadband and Wi-Fi, this proved challenging. Particularly in older thick-walled buildings, clinicians had to move rooms occasionally, and required installation of Wi-Fi boosters to get an adequate Wi-Fi signal to video consult. The additional new hardware increased the complexity of the process for clinicians, who were also asked to separately audio-record their consultations as Attend Anywhere did not permit simultaneous recording. Some patients’ broadband was insufficient for Attend Anywhere, which led occasionally to loss of contact during the consultations and a switch from VC to TC. Most patients used a smart phone for the consultations (21/43, 49%) or a computer/laptop (15/43, 35%). Three patients used 4G with variable success, but best results appeared to be with a fast Wi-Fi connection (frequencies of devices and internet connections used are available from the authors on request). Despite these problems, only three VC patients said that VC was not useful for dealing with their health problem. Recordings were generally of good quality and all were usable, though recordings of some TCs, particularly with mobile phones, were of lesser quality.

Of the planned 149 recorded consultations, 21 were changed to another consultation mode. Ten VCs changed to TC and four to FTFC; four planned TCs changed to FTFC and three planned FTFCs changed to TCs. Reasons for these changes were either patient or clinician choice or technology failure for the patient, the clinician, or both. If the consultation mode changed during the consultation (usually from VC to TC), the authors allocated it to the mode of consultation (VC or TC) with the longest recording for the purpose of RIAS analysis.

### Patient questionnaire

Of the 149 patients who provided a recorded consultation, 119 (80%) completed the online questionnaire exploring the technical quality and their general experience of the consultation. Patients reported that they generally found their consultation useful in addressing their problems, but FTFCs were scored ‘very good’ more frequently than TCs and VCs for all the GP Survey items: ‘doctor/nurse giving enough time,’ ‘asking about symptoms’, ‘listening’, ‘explaining tests and treatments’, ‘involving in decisions’, ‘treating with care and concern’, and ‘taking problems seriously.’

Responding patients who used VC were more likely to be working or in education: 34/43 (79%) (FTFC, 19/38 [50%]; TC, 16/38 [42%]) and more likely to feel confident using other types of internet communication: 28/43 (65%); (FTFC, 17/38 [45%]: TC, 16/38 [42%]); further details of work status and confidence in internet communication are available from the authors on request. Technical problems were more commonly reported with VC (14/43, 33%) than with TC (5/38, 13%). The biggest perceived advantages for patients of VC were convenience, including lack of need to travel or take time off work (Table 2). The main disadvantages were technical, with a small number who stated that they felt less cared for with a TC (3/38, 8%) and VC (3/43, 7%) than they would have with an FTFC (Table 3). Over 50% of patients in each of the TC and VC groups felt that there were no disadvantages to the mode of consultation (TC 21/38 (55%), VC 23/43 (53%) (Table 3). Responses to all patient questions are available from the authors on request.

**Table 2.** Responses to the instruction ‘*From the list of benefits of using telephone/video consulting, please tick all that apply to your consultation’* in patient questionnaire

**Response**	**TC, *n* (%) (*N*= 38)**	**VC, *n* (%) (*N*= 43)**	**Total, *n* (%) (*N*= 81)**
None selected	0	1 (2)	1 (1)
It saved travelling	31 (82)	36 (84)	67 (83)
Did not have to take time off work	13 (34)	17 (40)	30 (37)
More convenient	27 (71)	33 (77)	60 (74)
Because of my health condition, was safer/easier	4 (11)	4 (9)	8 (10)
Saved me money	2 (5)	5 (12)	7 (9)
Took less time	27 (71)	26 (60)	53 (65)
Saved me arranging childcare	0	1 (2)	1 (1)
Did not have to wait as long for an appointment	18 (47)	21 (49)	39 (48)
Other	1 (3)	6 (14)	7 (9)

Percentages are out of total number of patients for each consultation/overall. TC = telephone consultation. VC = video consultation.

**Table 3.** Patient responses to the instruction *‘From the list of disadvantages of using telephone/video consulting, please tick all that apply to your consultation’* in patient questionnaire

**Response**	**TC, *n* (%) (*N*= 38)**	**VC, *n* (%) (*N*= 43)**	**Total, *n* (%) (*N*= 81)**
None selected^a^	21 (55)	23 (53)	44 (54)
I could not hear the GP/nurse properly	4 (11)	7 (16)	11 (14)
I could not see the GP/nurse properly	—	7 (16)	—
It was a problem that the GP/nurse and I could not see each other	5 (13)	—	—
I could not find a private room to make the call	4 (11)	2 (5)	6 (7)
It cost me money	0	0	0
It was too complicated	0	0	0
My appointment took longer to arrange	3 (8)	1 (2)	4 (5)
I feel less cared for than if I had seen my GP/nurse in person	3 (8)	3 (7)	6 (7)
I needed an additional appointment	2 (5)	3 (7)	5 (6)
Other	6 (16)	4 (9)	10 (12)

a Three TC patients and two VC patients explicitly stated there were no disadvantages via the ‘Other’ option. TC = telephone consultation. VC = video consultation.

### Clinician questionnaire

Clinicians reported that VC appeared to be of less utility in managing patient problems: 36/42 (86%) found the FTFC to be very useful in managing the patients’ problems compared with 38/49 (78%) for TC and 26/40 (65%) for VC. When asked if they would choose this mode of consultation again for this presentation, 4/40 (10%) VC participants said they would not do so. However, this difference seemed largely owing to technical issues: 21/40 (52%) reporting some problems, and 6/40 (15%) reporting such a poor experience that they had to end the call. Further details of usefulness and technical qualities described by GPs for VC and TC are available from the authors on request.

### Consultation content

In general, patients raised fewer health problems in VCs and TCs on average than FTFC (mean 1.8 [SD 1.1] for TC and 1.5 [SD 0.8] for VC versus 2.1 [SD 1.1] for FTFC) and fewer problems were addressed on average during the course of VC compared with FTFC; further data on number of consultations by number of problems raised and addressed are available from the authors on request. The observed differences were particularly marked when VC was compared with FTFC; and indeed these differences were statistically significant for VC (*t*-test *P*<0.01), but not for TC (*P* = 0.12). FTFCs were longer on average (Table 4). FTFCs were 3.67 minutes (95% CI = 2.15 to 5.20) longer than VCs and 4.05 minutes (95% CI = 2.59 to 5.52) longer than TCs on average. FTFC, TC, and VC were similar in terms of the types of problems addressed; a table of types of problems by number of consultations is available from the authors on request.

**Table 4.** Consultation length (measured from recordings) by consultation mode

**Consultation length variable**	**Consultation mode^a^**		

	**FTFC (*n*= 51)**	**TC (*n*= 53)**	**VC (*n*= 45)**
Mean length, minutes (95% CI)	9.61 (8.34 to 10.89)	5.56 (4.81 to 6.31)	5.94 (5.15 to 6.73)
Median length, minutes (SD)	8.40 (4.53)	4.93 (2.72)	5.42 (2.63)
Minimum, minutes	2.33	1.45	1.45
Maximum, minutes	26.00	14.00	12.15

a Two patients recorded two consultations. CI = confidence interval. FTFC = face-to-face consultation. TC = telephone consultation. VC = video consultation.

### Consultation quality

Overall, the three consultation modes were mostly similar in terms of quality of care assessed using the RCGP framework (Table 5). TC and VC were similar, with some evidence of lower quality of consultations in two domains (seeking health understanding and placing problem in a psychosocial context) with differences evident when compared with FTFC. Lifestyle advice was given more often during the course of FTFCs and substantially less frequently in VCs (FTFC 22/50 [44%]; TC 12/49 [24%]; VC 6/43 [14%]). Broadly similar trends were observed in respect of the RIAS scoring framework based on clinician assessments, except in respect of rapport building, where some evidence of non-FTFC benefit was observed.

**Table 5.** RCGP quality indicators occurring at least once in each consultation mode

**RCGP indicator**	**Consultation mode, *n* (%)^a^**			

	**FTFC (*N*= 51)**	**TC (*N*= 53)**	**VC (*N*= 45)**	**Overall (*N*= 149)**
Patient’s own health understanding	31 (61)	22 (42)	17 (38)^b^	70 (47)
Patient concerns are sought	44 (86)	44 (83)	37 (82)	125 (84)
Places problem into psychosocial context	26 (51)	19 (36)	12 (27)^b^	57 (38)
Explanation of diagnosis	30 (59)	18 (34)^b^	18 (40)	66 (44)
Explanation of treatment	47 (92)	45 (85)	41 (91)	133 (89)
Checking understanding	51 (100)	48 (91)	43 (96)	142 (95)
Shared decision making	45 (88)	48 (91)	42 (93)	135 (91)
Safety netting	45 (88)	51 (96)	42 (93)	138 (93)

a Two patients recorded two consultations. Statistical significance calculated based on Fisher’s exact tests for TC or VC compared with FTFC:

b P<0.05. There were no significant differences between TC and VC. FTFC = face-to-face consultation. RCGP = Royal College of General Practitioners. TC = telephone consultation. VC = video consultation.

The RIAS showed that, in general, clinicians engaged in more patient education and counselling in FTFCs than both TCs and VCs. In turn, patients provided significantly more information in FTFCs (see Tables 6 and 7). There were no significant differences between VC and TC.

**Table 6.** Summary statistics for the number of occurrences of communication behaviour per consultation within each RIAS code grouping according to consultation type: clinician assessments

**Communication behaviour, mean (median, IQR)^a^**	**FTFC**	**TC**	**VC**
**Patient education and counselling**			
Provides biomedical information	45.16 (44, 27–57)	27.00^b^ (22,^b^ 9–36)	28.71^c^ (24,^c^ 11–42)
Provides psychosocial information	0.94	0.43	0.49
Counsels biomedical	10.18 (8, 1–15)	9.06 (8, 2–11)	9.24 (9, 3–14)
Counsels psychosocial	0.08	0.23	0.11
Total count	56.35 (54, 32–80)	36.72^c^ (30,^b^ 17–45)	38.56^c^ (38,^d^ 17–55)

**Data gathering**			
Open-ended biomedical questions	4.90 (4, 2–7)	3.47^d^ (3,^d^ 2–5)	3.89 (3, 2–5)
Closed-ended biomedical questions	7.43 (4, 2–9)	3.72^c^ (3, 1–5)	4.13^d^ (3, 2–5)
Open-ended psychological questions	0.14	0.32	0.18
Closed-ended psychological questions	0	0.17 (0,^d^ 0–0)	0.29 ^d^ (0,^d^ 0–0)
Bids for clarification	0.55 (0, 0–1)	0.53 (0, 0–1)	0.42
Total count	13.02 (10, 6–17)	8.21^c^ (8, 4–11)	8.91^d^ (7, 5–11)

**Rapport building**			
Personal remark	1.14 (0, 0–2)	2.81^c^ (1, 0–5)	3.24^c^ (2, 0–6)
Laughter/tells joke	0.49	0.17	0.18
Approval	0.02	0.19	0.04
Empathy	0.31	0.06	0.07
Legitimate	0.18	0.02 (0,^d^ 0–0)	0^d^ (0,^d^ 0–0)
Concern	0.39	0.25	0.11 (0,^d^ 0–0)
Reassure	0.80 (0, 0–1)	1.17 (0, 0–2)	0.80 (0, 0–1)
Total count	3.33 (2, 1–4)	4.66^d^ (4,^d^ 2–7)	4.44 (1–7)

**Partnership building**			
Paraphrase, checks understanding^e^	0.45	0.23 (0, 0–0)	0.16 (0,^c^ 0–0)
Verbal attention, shows partnership support	3.39 (1, 0–6)	2.21 (0, 0–5)	1.67 ^d^ (0,^d^ 0–2)
Asking clarification, bids for repetition	0.22	0.34	0.16
Asking clarification, asks for understanding	0.18	0.06	0.09
Asking clarification, asks for opinion	0.16	0.13	0.18
Total count	4.39 (3, 0–6)	2.96 (1, 0–5)	2.24^d^ (0, 0–3)

**Disagreement**			
Disagreement, shows direct disapproval	0.04	0	0
Disagreement, shows criticism in general	0	0	0
Total count	0.04	0	0

**Giving direction**			
Giving directions, transition, for example, request to allow examination	0.18	0 (0,^d^ 0–0)	0.09
Giving directions, gives orientation instructions, for example, go to examination couch	4.45 (3, 0–7)	2.87 (0, 0–5)	2.11^d^ (0,^d^ 0–4)
Total count	4.63 (3, 0–7)	2.87 (0, 0–5)	2.20^d^ (0,^d^ 0–4)

a *(Median, IQR) are (0, 0–0) if not shown. Statistical significance calculated based on two sample* t*-tests for TC/VC compared with FTFC (median tests are in brackets):*

b P*<0.001,*

c P*<0.01,*

d P*<0.05.*

e *There were no significant differences between VC and TC except for partnership building: paraphrase, checks understanding (median test* P*=0.02, higher frequency above the overall median for telephone. FTFC = face-to-face consultation. IQR = interquartile range. RIAS = Roter Interaction Analysis System. TC = telephone consultation. VC = video consultation.*

**Table 7.** Summary statistics for the number of occurrences of communication behaviour per consultation within each RIAS code grouping according to consultation type: patient assessments

**Communication behaviour, mean (median, IQR)^a^**	**FTFC**	**TC**	**VC**
**Patient education**			
Provides biomedical information	56.04 (44, 24–70)	30.72^b^ (25,^c^ 14–38)	27.02^b^ (22,^c^ 15–36)
Provides psychosocial information	4.10 (0, 0–6)	2.62 (0, 0–3)	2.67 (0, 0–3)
Total count	60.14 (49, 26–74)	33.34^b^ (30,^b^ 18–39)	29.69^b^ (23,^b^ 15–44)

**Data gathering**			
Open-ended biomedical questions	1.45 (1, 0–2)	0.64^d^ (0,^b^ 0–0)	0.69^d^ (0,^c^ 0–1)
Closed-ended biomedical questions	1.42 (1, 0–2)	0.85 (0, 0–1)	0.42^c^ (0,^d^ 0–1)
Open-ended psychological questions	0.08	0	0
Closed-ended psychological questions	0	0	0
Bids for clarification	0.14	0.49 (0,^d^ 0–0)	0.24
Total count	3.08 (2, 1–4)	1.98 (1, 0–2)	1.36^c^ (0,^b^ 0–2)

**Rapport building**			
Personal remark	0.92 (0, 0–1)	2.36^c^ (1, 0–4)	2.22^c^ (0, 0–5)
Laughter/tells joke	0.43	0.38 (0, 0–1)	0.38
Approval	0.04	0.75^c^ (0^b^, 0–1)	0.29^d^
Empathy	0	0	0
Legitimate	0	0	0
Concern	0.16	0.09	0
Reassure	0.14	0.04	0.04
Total count	1.69 (1, 0–3)	3.62^b^ (3,^b^ 2–5)	2.93^d^ (2, 0–5)

**Partnership building**			
Paraphrase, checks understanding	0.10	0.06	0.02
Verbal attention, shows partnership support	0.39	0.02	0.16
Asking for clarification, bids for repetition	0.02	0.30^d^ (0^c^, 0–0)	0.16
Asking for clarification, asks for understanding	0	0.13	0.02
Asking for clarification, asks for opinion	0.12	0.06	0.07
Total count	0.63	0.57	0.42

**Disagreement**			
Disagreement, shows disapproval direct	0.02	0	0
Disagreement, shows criticism general	0	0	0
Total count	0.02	0	0

**Giving direction**			
Giving directions, transition	0	0	0
Giving directions, gives orientation instructions	0	0	0.04
Total count	0	0	0.04

a (Median, IQR) are (0, 0–0) if not shown. Statistical significance calculated based on two sample t-tests and median tests for TC/VC compared with FTFC:

b P*<0.001,*

c P*<0.01,*

d P*<0.05.*

FTFC = face-to-face consultation. IQR = interquartile range. RIAS = Roter Interaction Analysis System TC = telephone consultation. VC = video consultation.

### Impact on subsequent workload

Records were searched to determine if patients were more or less likely to have a follow-up appointment after the index consultation. Around half of the patients contacted the surgery in the subsequent 4 weeks (further details of the frequency of contact are available from the authors on request). Consultation frequencies were similar across the three modes of consultations. A similar proportion of subsequent consultations were for follow-up of the index consultation.

## DISCUSSION

### Summary

In terms of content, VC appeared similar to TC in dealing with a lower number of problems than FTFC and having a shorter consultation time. RIAS data revealed a much richer consultation in FTFCs in terms of information provision and advice given than both VC and TC, though VC may offer advantages in respect of building rapport. Overall, patient experience appeared better in FTFC than both VC and TC, which were similar.

VC was taken up by mainly younger, technically informed people, who had consulted less often than those opting for FTFC. When considering content and quality of the clinician–patient interaction, VC is similar to TC, both address fewer problems, and contain less exchange of information in comparison with FTFCs. Implementing VC technology in NHS practices was challenging and a fully integrated system will require infrastructural improvements to many surgeries. However, patients value its convenience and, where physical examination is not required, it may offer advantages over both FTFC and TC.

### Strengths and limitations

Although others have explored attitudes to VC in primary care^20^^,^^21^ and its use in other healthcare settings,^22^ to the authors’ knowledge this is the first in-depth analysis of the actual use of VC in primary care. Given the recent controversial introduction into general practice of privately run VC,^9^ and the seeming reluctance of most GP practices to introduce it,^23^ the present study provides timely, important evidence of how VC may differ from FTFC and TC in terms of content, quality, and patient experience.

This study has several limitations regarding the type of consultation studied and self-selection by patients. The researchers focused on follow-up consultations in this study for several reasons as they considered it would be easier for clinicians to assess the need for a physical examination, which would require a FTFC. Additionally, previous research in telephone consulting has shown that patients and clinicians feel more comfortable with a remote consulting medium when the condition has already been diagnosed^2^ and where they already know and have met the GP. Given the novelty of VC for both patients and clinicians, the authors felt it was preferable to provide what might be considered a less risky consultation. Finally for practical reasons clinicians thought that it would be difficult for reception staff to determine eligibility, explain the study, and how the system worked for initial presentations. One advantage the authors hoped from restricting participation to follow-up consultations was a reduced heterogeneity of the sample to facilitate comparison between consultation modes.

Patients self-selected the mode of consultation. Clinicians found it hard to recruit patients to VC and those who agreed to this were younger. Although the problem types encountered were similar across the three modalities, it may be that patients decided that what they felt were complex consultations would be better dealt with face-to-face and, potentially, clinicians may also have influenced this. Patients who chose FTFCs discussed more problems. It is not clear if this was because the consultation type facilitates raising additional problems or if patients knew in advance they had several problems to discuss and deliberately chose FTFC. However, randomised trials of same-day requests for consultations show that TCs deal with fewer problems, and qualitative research has found that patients and clinicians have an expectation that TCs are for single uncomplicated problems and perhaps VC was regarded similarly.^2^^,^^3^^,^^24^ Still, this was a patient population’s first ever experience with video consulting and it may be that, with experience, consultations may change. For patients who work or are housebound the medium proved very popular, but it was generally a younger (possibly healthier) working population who took it up.

Interviews with patients and clinicians (who could be described as enthusiastic early adopters), reported in the authors’ companion article,^14^ showed that in general they thought that, when the technology works, VC has advantages over TC regarding the rapport achieved and in facilitating understanding through non-verbal communication. It was considered particularly useful in consultations involving psychological assessment where visual cues are important, but physical examination is unnecessary.

### Comparison with existing literature

Recent research on VCs in the secondary care sector in the UK^21^ showed similar findings with shorter consultations and similar problems with technology. Uptake of VC in the hospital setting was low, and, similarly, most of the clinicians (who were enthusiastic) also had difficulty in recruiting patients to take part. Similar findings with regard to uptake and technology problems have been found in international studies.^25^^–^27 However, GP at Hand,^9^ a commercially delivered, video-based primary care service in London, which comes with guarantees of immediate access, has had a large number of patients joining and the company claim high satisfaction rates, admittedly from a relatively young and healthy population.

Though the present research demonstrates the limitations of VC compared with FTFC to an extent, others have argued that, even if the outcome of VC is inferior to that of FTFC, it can be within the acceptable range of validity for clinical purposes and, considering the convenience of remote consultation, VC or TC could still be the preferred mode of consultation.^28^

### Implications for research and practice

Implementation theory^29^ suggests that, to be successful, any VC system must be simple to use, be seen to be an advance over existing technology, and ideally integrate with existing work patterns and surgery computer systems. Considerable work may be required to integrate VC with current NHS systems to meet the year target set by the *NHS Long Term Plan* and this may have resource implications.^7^ Though patients liked VC the advantages to clinicians are less clear, such as overall workload. VC has a similar duration, content, and impact on follow-up consultations as TC. Additionally, the reduction of some elements of care, particularly information giving, and consultation length may have consequences for overall experience and effectiveness of care compared with FTFC, and may also have implications for other parts of the health service.

Before or during implementation, further research is urgently needed to determine the best role for VC in terms of suitability for patients and clinical conditions, and the risks and benefits associated with it. Ideally a randomised controlled trial should be conducted to explore the differences between VC and other modes of consultation, its impact on resources, and including use in first presentations, preferably in practices where VC is more established.
